# Production and Characterization of New Biosurfactants/Bioemulsifiers from *Pantoea alhagi* and Their Antioxidant, Antimicrobial and Anti-Biofilm Potentiality Evaluations

**DOI:** 10.3390/molecules28041912

**Published:** 2023-02-17

**Authors:** Badiaa Essghaier, Nesrine Mallat, Khaoula Khwaldia, Filomena Mottola, Lucia Rocco, Hédia Hannachi

**Affiliations:** 1 Laboratory of Mycology, Pathologies and Biomarkers LR16ES05, Faculty of Sciences of Tunis, University of Tunis El Manar II, Tunis 2092, Tunisia; 2 Laboratoire des Substances Naturelles, Institut National de Recherche et d’Analyse Physico-Chimique (INRAP), BiotechPole, Sidi Thabet 2020, Tunisia; 3 Department of Environmental Biology and Pharmaceutical Sciences and Technologies (DiSTABiF), University of Campania L.Vanvitelli, 80100 Caserta, Italy; 4 Laboratory of Vegetable Productivity and Environmental Constraint LR18ES04, Department of Biology, Faculty of Sciences, University Tunis El-Manar II, Tunis 2092, Tunisia

**Keywords:** biosurfactants, bioemulsifiers, glycolipid, *Pantoea alhagi*, antioxidant, antimicrobial, anti-biofilm

## Abstract

The present work aimed to develop rapid approach monitoring using a simple selective method based on a positive hemolysis test, oil spreading activity and emulsification index determinations. It is the first to describe production of biosurfactants (BS) by the endophytic *Pantoea alhagi* species. Results indicated that the new BS evidenced an E24 emulsification index of 82%. Fourier-transform infrared (FTIR) results mentioned that the described BS belong to the glycolipid family. Fatty acid profiles showed the predominance of methyl 2-hyroxydodecanoate in the cell membrane (67.00%) and methyl 14-methylhexadecanoate (12.05%). The major fatty acid in the BS was oleic acid (76.26%), followed by methyl 12-methyltetradecanoate (10.93%). Markedly, the BS produced by the *Pantoea alhagi* species exhibited antimicrobial and anti-biofilm activities against tested human pathogens. With superior antibacterial activity against *Escherchia coli* and *Staphylococcus aureus,* a high antifungal effect was given against *Fusarium* sp. with a diameter of zone of inhibition of 29.5 mm, 36 mm and 31 mm, obtained by BS dissolved in methanol extract. The DPPH assay indicated that the BS (2 mg/mL) showed a higher antioxidant activity (78.07 inhibition percentage). The new BS exhibited specific characteristics, encouraging their use in various industrial applications.

## 1. Introduction

Natural sources of surfactants or biosurfactants (BS) are surface active amphiphilic compounds of biological origin. A large variety of microorganisms, such as bacteria, fungi and algae, are efficient at producing biosurfactants [1]. BS offer many advantages compared to synthetic ones; they are environmentally more compatible than chemically synthesized surfactants. They provide meaningful properties such as bioavailability, biodegradability, activity under extreme conditions, low toxicity and structural diversity [2]. Based on their biochemical structures, BS are divided into five major classes, namely, lipopeptides (LPs), glycolipids, lipoproteins, phospholipids and polysaccharides [3,4]. In general, BS reveal diverse properties and can be used as (i) bioemulsifiers (BE), which can reduce surface and interfacial tension between immiscible liquids by making mixtures of different polarities compatible, and (ii) antibiotics and antifungal agents [5]. BS also manifest antimicrobial properties by directly disrupting the cell wall of Gram-positive and Gram-negative bacteria or disruption of bacterial biofilm, e.g., as shown in [6]. Despite the presence of numerous studies on the production of BS, the discovery of new microbial surfactant properties is of great purpose. BS offer diverse applications in different fields close to industry, environment protection, pharmacy and medicine, food and cosmetics [7,8], agriculture [9] and bioremediation [10]. Therefore, greater attention has been paid to BS and their future applications. In this context, several studies were performed to explore new applications of BS [9]. Hence, extensive efforts have been made to isolate and characterize new BS. These BS have advantages, such as low toxicity and high selectivity action at extreme pH and temperature, over their synthetic counterparts [1]. In fact, bacteria are able to modify the lipid composition and organization of the cell membrane as adaptation mechanisms to environmental factors and stress, and, thus, exogenous fatty acids confer protection against membrane stress [11]. In Gram-positive bacteria, the predominant phospholipids are phosphatidylglycerol (PG) and cardiolipin (CL) [12]. The composition depends on the growth condition and the bacterial species [13].

Extremophiles are microorganisms that live in extreme conditions. To adapt to these hostile conditions, they have developed biological mechanisms. Therefore, extremophiles have gained the attention of several researchers who aimed to search for and discover new biomolecules relating to various biotechnological applications [14]. Among these, BS are the most used compounds for industrial processes in industries such as the food, cosmetic and waste cleaning industries [15].

Based on the explanation above, the present work is the first one that aims to identify the fatty acid composition of the cell membrane of a new isolate belonging to the *Panteoa alhagi* species and to evaluate the specific fatty acid composition of the BS synthesized by this bacterial strain.

To our knowledge, there have been no reports describing the production of BS by the *Panteoa alhagi* species. In the present work, we attempt the isolation of a new bacterial strain producing BS from the desert located in Southern Tunisia which has BS potentialities. Investigation of the production, characterization and antimicrobial and antioxidant capacities of the BS is undertaken.

## 2. Results

### 2.1. Bacterial Identification

**Figure 1:**
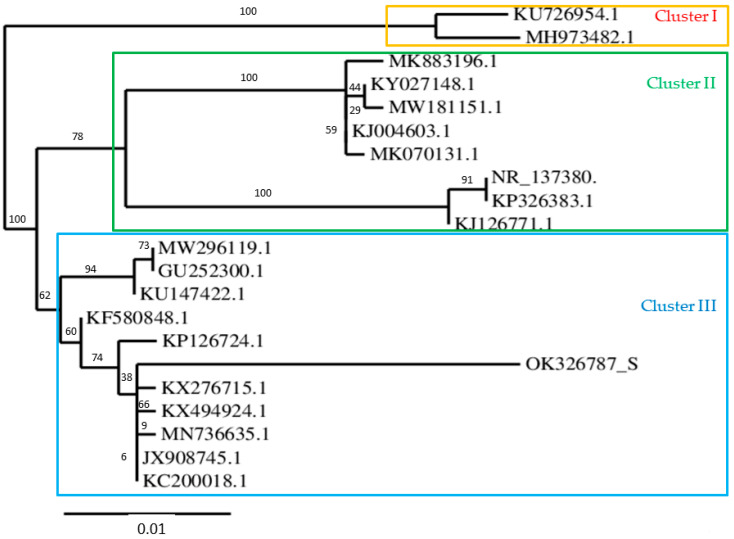
Neighbor-joining phylogenetic tree based on 16S rRNA gene sequence showing the taxonomical relation of *Panthoa alhagi* strain Zcb15 (accession number: OK326787) and other bacterial strain sequences deposit in GenBank. Bootstrap values are based on 1000 repetitions. GenBank accession numbers of sequences used: K537297.1; KJ126907.1; GU252300.1; EF088378.1; KF580848.1; KP126724.1; KU147422.1; MK070131.1; KF600442.1; MW181151.1; KY027148.1; NR_137380.1, KP326383.1; KJ004603.1; KJ126771.1; MK883196.1; KU726954.1; KY194258.1; MH973482.1; JX908745.1; KC200018.1; KX494924.1; KX276715.1; MN414603.1; MW296119.1; GU252300.1; MN736635.1. Bar 0.01 substitutions per nucleotide position.

From a list of halophilic bacteria isolates, in this work, we selected the producer of the most biosurfactants, the strain Zcb15, a new halophilic bacterial isolate from an extremophile plant (*Zygophyllum cornarum* species) located in Southern Tunisia (arid region). The bacterial isolate is a rod-shaped, Gram-negative bacterium, which is oxidase negative and catalase positive. The sequencing and phylogenetic analysis of the 16S rRNA gene were performed. The associated 16S rDNA sequence was deposited in GenBank (accession number: OK326787). BLAST tools analysis demonstrated an identity of 97.39% with *Pantoea alhagi* strain NX-11 (MN736635.1). The phylogenetic analysis also placed the strain Zcb15 in cluster III, which contains *Pantoea alhagi* (MW296119.1 and MN736635.1), *Pantoea sp* LTYR-11Z (KX494924.1) and *Erwinia amylovora* strain KYU90 (GU252300.1) (Figure 1).

### 2.2. Biosurfactants Production

Both qualitative and quantitative methods were used to detect the production of BS by the tested strain Zcb15.

#### 2.2.1. Hemolytic Activity

**Figure 2:**
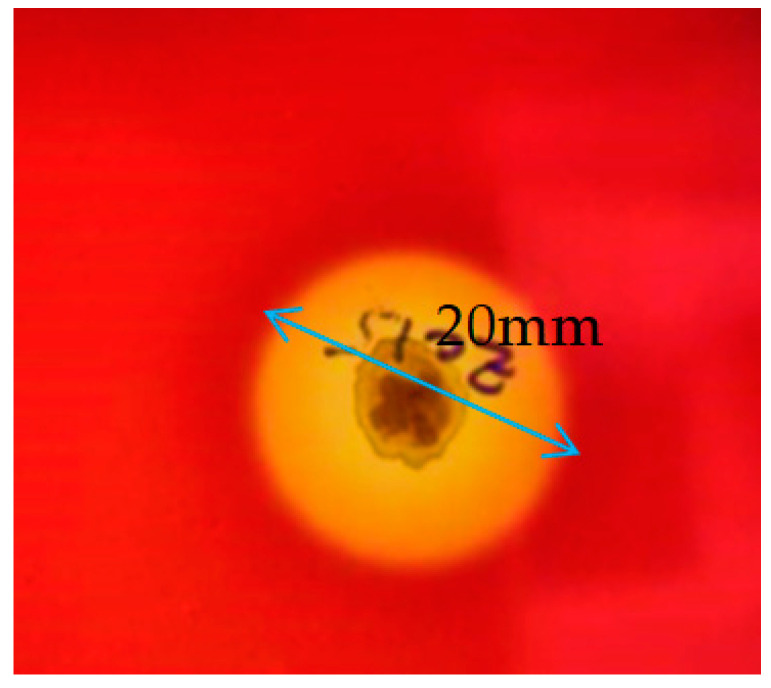
Hemolysis test results confirmed the presence of biosurfactants of the glycolipid family, as shown by the clear zone around the bacteria colony of *Pantoea alhagi* strain Zcb15.

Visual observation of the transparent zone around the Zcb15 strain colony on the blood agar plates, the lysis method (a clear zone of 20 mm), indicated the production of BS of the glycolipid type based on the hemolysis test, as described in [16]. Moreover, the hemolytic test confirmed that the diameter of the clear zone was proportional to the glycolipid secretion (Figure 2).

#### 2.2.2. The Oil Spreading Test

**Figure 3:**
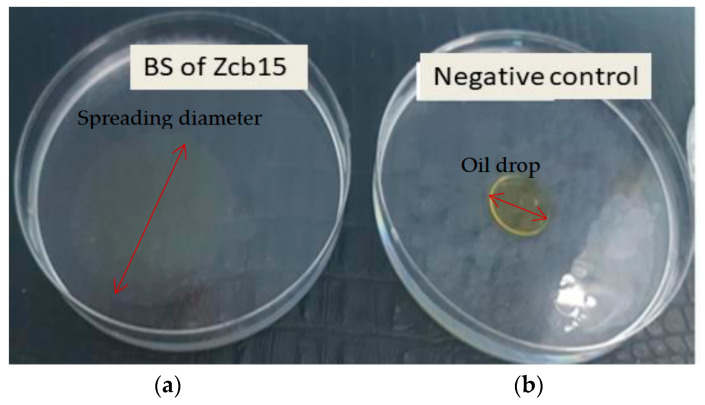
Oil spreading method observed in the Petri dishes of a sample containing the biosurfactant solution of strain Zcb15 (**a**) as compared to the negative control (distilled water and oil) (**b**).

Oil spreading is a simple and rapid method used to detect the production of BS. This method is based on the examination of water–oil interfacial tension reduction and the expansion of a clear zone due to the presence of effective biosurfactants (spreading diameter of the oil drop compared to control one) (Figure 3).

#### 2.2.3. The Emulsification Activity

**Figure 4:**
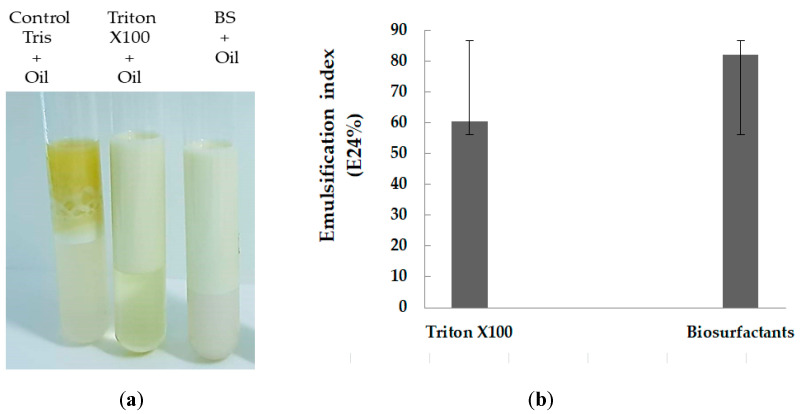
Emulsification index (E24) observed in the presence of BS. The cell-free supernatant of *Pantoea alhagi* strain Zcb15 as compared to the presence of Triton X-100 as positive control and Tris base buffer as negative control (**a**) and the E24 index of the BS secretion by the strain Zcb15 expressed in a percentage as compared to the positive control (Triton X-100) (**b**).

The strain Zcb15 of *Pantoea alhagi* was able to emulsify the olive oil, which was shown by a clear emulsified layer (Figure 4a). The emulsification activity of the cell-free broth culture was measured against olive oil and compared to the positive emulsifier (Triton X-100) and negative control. The E24 indices were calculated, and the results proved the superior activity of BS produced by strain Zcb15, as indicated by the E24 emulsification index assays of about 82% on the LB medium (Figure 4b).

### 2.3. Biosurfactants Production Optimization

The highest production of BS by strain Zcb15 was observed in the presence of LB medium in addition to sunflower oil as a single carbon source compared to different vegetable oils tested in this work. Sunflower oil was selected as the superior inducer of secretion of BS by the studied bacterial strain (Table 1).

### 2.4. FTIR Analysis

**Figure 5:**
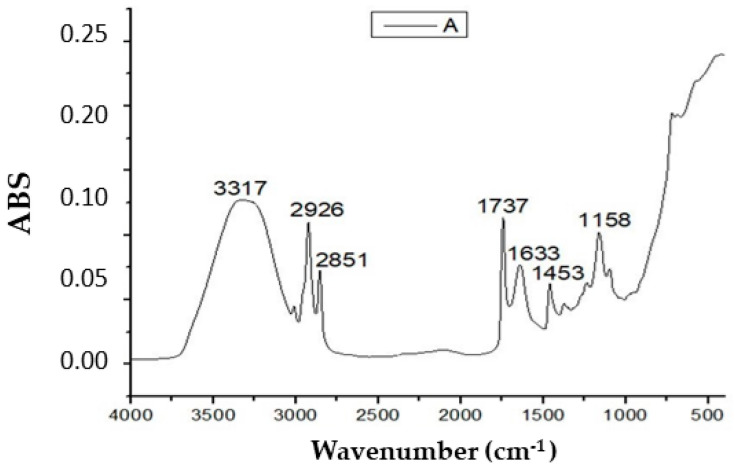
Fourier-transform infrared (FTIR) analysis of BS produced by *Pantoea alhagi* strain Zcb15.

The characteristic peak at 3317 cm^−1^ indicated the presence of primary or secondary amines (N–H stretching group). The peaks at 2926 and 2851 cm^−1^ corresponded to C–H aliphatic stretching. The peak at 1737 cm^−1^ was relevant to the vibration of the C=O of the ester, and the peak at 1633 cm^−1^ corresponded to mono-substituted C=C stretching. The peak at 1453 cm^−1^ was assigned to the O–H bend of carboxylic acid. Moreover, the FTIR spectrum of the BS revealed the presence of an absorption peak at 1158 cm^−1^ that may be attributed to C–O stretching (aliphatic ether). Based on the functional groups detected by the FTIR analysis, the biosurfactants produced by strain Zcb15 have a structure identical to that of glycolipids, as previously described [17] (Figure 5).

### 2.5. Fatty Acid Analysis

#### 2.5.1. Fatty Acid Composition of Biosurfactants and Cell Membrane of *Pantoea alhagi* Strain Zcb15

**Figure 6:**
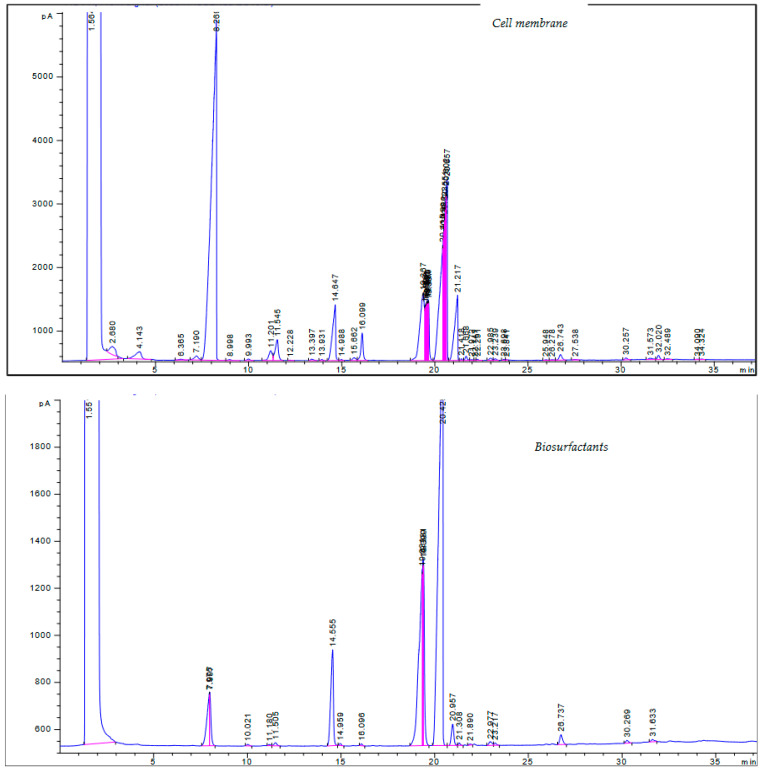
Chromatogram profiles of cell membrane and fatty acid compositions of biosurfactants of *Pantoea alhagi* strain Zcb15.

The chromatogram profile allowed the identification of 22 fatty acids in the cell membrane of bacterial strain Zcb15 of *Pantoea alhagi* (see Figure 6 cell membrane) and 13 in the product BS ( see Figure 6 Biosurfactants). Therefore, the production of the BS lost nine compounds (Figure 6).

Methyl 2-hyroxydodecanoate was the major compound of the cell membrane, amounting to 67.00%, followed by methyl 14-methylhexadecanoate, with an amount of 12.05%. The produced BS showed that oleic acid was the major compound (76.26%), followed by methyl 12-methyltetradecanoate (10.93%) (Table 2)

#### 2.5.2. Fatty Acid Comparison of Biosurfactants

Table 3 shows the fatty acid profiles in the BS produced by the strain Zcb15 of *Pantoea alhagi* and BS from other bacterial strains. Results markedly indicated that, qualitatively and quantitatively, the fatty acid composition varied between each biosurfactant related to the bacterial species and strains. The BS produced by strain Zcb15 of *Pantoea alhagi* were the most rich in oleic acid (C18:1n-9), with an amount of 76.26%.

### 2.6. Antimicrobial and Anti-Biofilm Potentialities of the Biosurfactants

**Figure 7:**
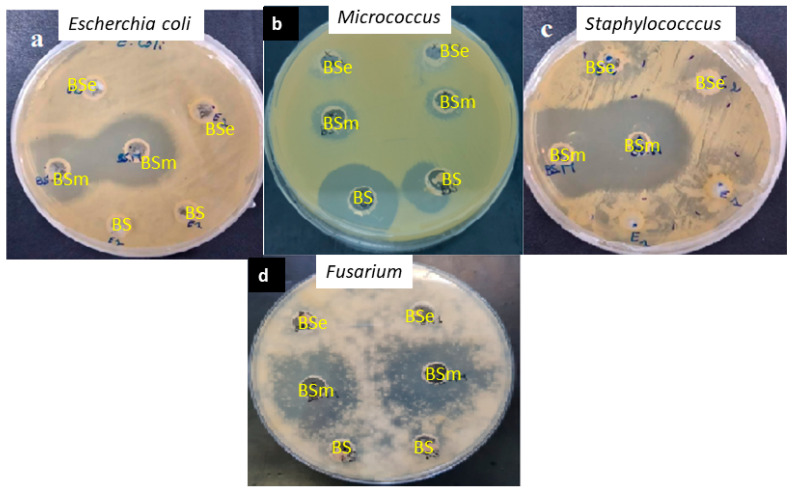
The antimicrobial activities of the biosurfactant pellet (BS) at 2 mg and the BS solution dissolved in methanol (BSm) and acetate ethyl (BSe) at 2 mg.mL^−1^, respectively, against human pathogens. With (**a**): *Escherchia coli*; (**b**): *Micrococcus luteus*; (**c**): *Staphylococcus aureus* and (**d**): *Fusarium sp*.

The BS pellet (2 mg) was able to inhibit the pathogens *Micrococcus luteus* and *Fusarium* sp. with a zone of inhibition diameter of 21.75 and 15.5 mm, respectively. In addition, the dissolution of BS in methanol and ethyl acetate at 2 mg.mL^−1^ improved their antimicrobial activities, as illustrated by the increase in the zone of inhibition detected (in mm) against pathogen strains (Table 4). The greatest zone of inhibition was observed for the BS dissolved in methanol with 36 mm and 31 mm against *Staphylococcus aureus* and *Fusarium sp*., respectively (Figure 7). Methanol enhanced the antibacterial effect of the BS. No antimicrobial action was observed with the methanol or ethyl acetate alone against the pathogen by the agar well diffusion method.

The BS also revealed an anti-biofilm activity against the biofilm of *Micrococcus luteus* and *Fusarium* sp. with an inhibition percentage of 58.35 and 77.28, respectively.

### 2.7. Antioxidant Activity

The BS secreted by strain Zcb15 showed high antioxidant potential by DPPH assay; the antioxidant activity was dose dependent on the BS in methanol solution. The maximum observed was at 2 mg.mL^−1^, with 78.07 percentage of inhibition (Table 5).

## 3. Discussion

In the last few decades, natural polymers have gained much attention in scientific research. Endophytic bacteria are sources of novel biomolecules for biotechnological purposes. It is an agreeable and rapid strategy to target plant microbiota, which confer resistance and adaptation to harsh environmental conditions (toxic metal, abiotic and biotic stress) [22,23]. Extremophiles are microorganisms that require extreme conditions to grow optimally; thus, to live there, they have developed biological adaptive mechanisms showing attractive characteristics for biotechnological applications. Extremophiles are markedly targeted in the search for and discovery of new bioproducts [14]. Surfactants are considered one of the most targeted bioproducts by different industrial sectors, such as the pharmaceutical, food, cosmetics and toxic waste cleaning industries [24].

In this context, we chose the halophyte plant *Zygophyllum cornutum*, located in Southern Tunisia (arid region, contaminated soil), to isolate new exopolysaccharides (EPS)-producing bacteria which probably possess beneficial properties. EPS play a prominent role against pathogens, toxic compounds and osmotic stress, and more attention has been directed to their antimicrobial, anti-biofilm and antitumor potentials [25]. In fact, EPS-producing microorganisms isolated from several extreme environments have acquired biotechnological applications. Moreover, some EPS are employed as BS [26]. Recently, in 2017, the endophytic species *Pantoea alhagi*, as a producer of EPS, was isolated and identified [27].

Here, we firstly described the BS produced by the strain Zcb15 of *Pantoea alhagi*. It is reasonable to hypothesize that the isolation and the identification of new microorganisms from extreme environments will provide new metabolites exhibiting novel properties for biotechnological purposes. Moreover, several studies showed that BS are usually effective in extreme conditions. In the present research, the new strain Zcb15 was isolated and identified. Its production of BS was examined, and the BSs were characterized based on multiple screening methods, including a hemolytic activity test, the oil spreading technique and emulsification index (E24). In general, the screening methods used for biological surfactant detection include drop collapse, oil displacement, hemolysis tests and emulsification index (E24) [28].

In the present research, the hemolysis test and the emulsification index, as well as the oil spreading test, were used to select the bacteria that were the best producers of BS. Several works explained that the diameter of the zone observed in the hemolysis technique is related to the secretion of BS. It is well known that there are many biomolecules that can cause red blood cell lysis by the hemolytic method but do not necessarily have the properties of surface active molecules (BS) [29]. On the other hand, this method specifies the glycolipid type of the BS produced by the bacteria. These findings were confirmed by FTIR analysis. The best-known glycolipids are surfactin, rhamnolipids, sophorolipids and trehalolipids [30].

The obtained emulsification index of 82% was higher than that reported for other bacterial species such as *Bacillus*, which gave an E24 of 65% in the presence of olive oil. The production of BS is influenced by the carbon sources [31]. Results showed that the strain Zcb15 displayed the best production of BS in the presence of sunflower oil; similar enhancement of production of BS by this oil was also obtained for *Bacillus* strain BK34, as reported previously [31]. Thus, superior emulsification is also critical for exploitation of BS in environmental or industrial applications [32,33]. Nino et al. [32] described that the composition of the medium (carbon and azote sources) and emulsifying action were related to the microbial origin. Numerous studies have described the direct correlation between oil displacement area, drop collapse and surface tension assays [34,35,36].

Results of the fatty acid analysis of the cell membrane of strain Zcb15 of *Pantoea alhagi* were distinguishable from those observed in strains phylogenetically closely related, for example: C12:0 (0.46%) compared to *Pantoea alhagi* strain LTYR-11ZT and *Pantoea theicola* strain NBRC 110557T and *Pantoea intestinalis* DSM 28113T, which showed more than 4.2%. The strain Zcb15 was marked by a lack of C14:0, unlike the other strains of *Pantoea*, which contain more than 6.5%. For C13:0, our strain Zcb15 of *Pantoa alhagi* contained a value of 0.09, which is near to the value observed in strain LTYR-11ZT of *Pantoea alhagi*. Strain Zcb15 contained C16:0 at 0.06%, as opposed to both strains *Pantoea theicola* strain NBRC 110557T and *Pantoea intestinalis* DSM 28113T, which showed the absence of C16:0. The obtained value of C18:0, with an amount of 1.72%, was near to that observed in *Pantoea theicola* strain NBRC 110557T, which had 1.2% [27].

Considering the antibiotic resistance emergence in pathogenic bacterial species, researchers pointed out antibiotic alternatives such as antimicrobial peptides and lipopeptides [27,37]. Here, the strain Zcb15 was able to produce BS showing antimicrobial and anti-biofilm potentialities. It has been reported that the BS produced by *Bacillus* species also have antibacterial action [27,38]. The current BS produced by *Pantoea alhagi* exhibited superior antimicrobial effect, with a ZI ranging from 13.75 to 36 mm as compared to those previously described [39], where ZI values did not exceed 9.64 mm.

It is well known that biofilm colonization offers adaptation and resistance of microorganisms, and it presents one of the factors of virulence; as a result, BS showing an anti-adhesive action could be an effective strategy applicable to reduce microbial adhesion to solid surfaces [40]. For example, the glycolipid produced by *Burkholderia* sp. strain WYAT7 has shown antibacterial and anti-biofilm potentialities [41]. BS have also proven their great inhibitory effect on microbial adhesion and biofilm formation [40,42]. On the other hand, the current work found that BS from strain Zcb15 were able to inhibit growth and biofilm formation of plant fungi *Fusarium* sp.; this finding promotes their use in agriculture fields. In the same context, Araujo et al. [10] demonstrated that the BS from *Serratia marcescens* can enhance the seed germination for agricultural application and oil spill bioremediation.

Markedly, the biosurfactant produced by *Pantoea alhagi* strain Zcb15 showed antioxidant potential. Several works described the antioxidant potential of BS, for example, the BS of *Bacillus subtilis* RW-1, which showed an inhibition percentage of 80.6 in a BPPH assay [43]. Thus, the BS can be used as reducing agents in food formulations. It should be noted that the antioxidant potential is related to the structure of the BS.

Here, we propose the application of BS of microbial origin as ecofriendly agents in remediation technologies and for enhancing oil recovery in agricultural chemicals [31].

## 4. Materials and Methods

### 4.1. Bacteria Isolation and Phylogenetic Identification

The bacterium isolate of *Pantoea alhagi* strain Zcb15 used in this work was isolated from the extremophile plant belonging to *Zygophyllum cornarum* species located in Southern Tunisia (desert region). The isolation was undertaken on nutrient agar with 5% added sucrose to induce EPS production [44]. The morphological and biochemical characterizations were verified according to the standard biochemical tests as recorded in *Bergey’s Manual of Systematic Bacteriology*. The 16S rDNA was PCR amplified using the universal primer pair Fd1 (5′AGAGTTTGATCCTGGCTCAG3′) and S17 (5′ GTTACCTTGTTACGACTT3′). PCR reaction was performed in 25 µL volume containing 1X buffer, 0.2 µmol L^−1^ of each primer, 0.2 mmoL dNTPs and 0.2 Taq DNA polymerase (Sigma-Aldrich, St. Louis, MO, USA) and 25 ng of template DNA. The PCR conditions consisted of initial denaturation at 94 °C for 5 min followed by 35 cycles of denaturation at 94 °C for 30 s, an extension at 72 °C for 45 s and a final extension at 72 °C for 7 min. The rDNA 16S sequence was deposited in the GenBank with accession number OK326787. The gene sequence was analyzed by BLAST, and the phylogenetic tree was constructed using ClustalW2. The culture was maintained on nutrient agar slants at 4 °C until use.

### 4.2. Biosurfactants Production

#### 4.2.1. Hemolytic Activity

The bacterial strain was incubated on the surface of blood agar medium containing 5% sheep blood for 24 h to 48 h at 37 °C. The observation of the clearing zone around the bacterial colony indicated the secretion of BS of the glucolipid type by the hemolysis activity [45].

#### 4.2.2. Oil Spreading Test or Oil Displacement Test

Approximately 500 µL of vegetable oil was deposed on the water surface in Petri dishes, and 500 µL of cell free supernatant was gently put on the oil surface. The diameter of the clearing zone was measured. Triton X-100 was used as positive control and olive oil as negative control [46].

#### 4.2.3. Emulsification Index (E24)

The emulsification index E24 was estimated by mixing equal volumes (V/V) of cell-free supernatant and vegetable oil in a test tube and mixed with a vortex at maximum speed for 2 min. The emulsification index E24 was determined after 24 h, and it was calculated as the ratio of the height (h) of the emulsion layer to the total height of liquid (mm) as follows [47]:

E24 (%) = h emulsification/h total × 100

#### 4.2.4. Emulsification Assay

In test tubes, 1 mL of cell-free supernatant was added to 5 mL of Tris buffer (50 mM, pH 8.0) and 5 mL of the vegetable oil. The mixture was vortexed for 2 min, and the emulsion mixture was allowed to settle for 20 min at room temperature. The optical density was measured at 400 nm. The emulsification activity per mL was calculated based on the following formula:

1 Emulsification Unity (UE) = 0.01 OD400 ×dilution factor

A negative control consisted of a Tris buffer solution and crude oil. The positive control used was Triton X-100 [48].

#### 4.2.5. Carbon Source Effects on Biosurfactants Production

In order to test the effect of carbon source on the production of BS by the strain Zcb15, Tween 80, olive oil, corn oil and sunflower oil were added separately to the LB medium at 30 °C for 72 h with agitation at 130 rpm. The samples were then centrifuged at 8000 rpm/min at 4 °C for 10 min, and the supernatant was recuperated to measure the OD at 400 nm as described above in Section 4.2.4.

### 4.3. Fourier-Transform Infrared (FTIR) Analysis of Biosurfactants

The FTIR spectrum analysis of BS was carried out on an FTIR Bruker Equinox 55 spectrometer (Bruker Co., Ettlingen, Germany) using an attenuated total reflection (ATR) accessory with a diamond ATR crystal. A total of 32 scans were performed at 4 cm^−1^ resolution. Measurements were recorded between 4000 and 600 cm^−1^ [49].

### 4.4. Extraction of Biosurfactants

Strain Zcb15 was cultivated on 150 mL LB medium supplemented with sunflower oil after incubation at 30 °C for 72 h. The culture was centrifuged at 8000 rpm at 4 °C for 30 min to separate the supernatant from medium solution. The supernatant was recovered and acidified by 1N HCl until pH 2 and then incubated overnight at 4 °C. The pellet of BS was obtained after centrifugation at 8000 rpm for 30 min at 4 °C [50].

### 4.5. Fatty Acid Analysis of Cell Membrane of Pantoea Alhagi Strain Zcb15 and Its Biosurfactants

After growing on LB medium at 30 °C for 48 h, the bacterial colonies of strain Zcb15 were recuperated from the surface in a sterile tube. The biosurfactants were extracted based on the method detailed above in Section 4.4. 

Whole fatty acids from both samples (cell membrane and BS) were saponified, methylated and extracted according to the method of MIDI microbial identification system (version 6.0) as previously described [51].

Fatty acid methyl esters were analyzed by GC (6890N, network GC system (Agilent technologies, Hewlett Packard), and the TSBA6 database of the identification system was used [27].

### 4.6. Antimicrobial Acticity of BS

#### 4.6.1. Agar Well Diffusion Method

The BS pellet obtained above was dissolved in various organic solvents (acetate ethyl or methanol). Human pathogen strains (*Staphylococcus aureus*, *Micrococcus luteus*, *Escherichia coli*) and plant pathogen fungi (*Fusarium oxysporum*) were used. For that, 100 µL of microbial pathogen suspension adjusted to 10^6^ CFU mL^−1^ was used and spread onto the surface of the agar plate medium. Then, 40 µL of BS solution at 2 mg.mL^−1^ was put on the well. The observation of the clear zone around each well indicated the presence of antimicrobial activity. Methanol and acetate ethyl were used as negative control [52].

#### 4.6.2. Anti-Biofilm Activity

To detect the effect of the BS on the biofilm formation of the pathogenic species, the surface of 96-well, flat-bottomed microtiter plates was used [53]; for that, 200 µL of 10^6^ cells mL^−1^ PBS was added to the wells and incubated at 37 °C for 24 h. The non-adherent cells were removed by washing with PBS, then, after, 40 µL of the BS solution was added and incubated at 37 °C for an additional 24 h. Negative control wells received only 200 µL of PBS. Crystal violet (20 µL) at 0.1% (w/v) was added for 15 min to quantify the microbial biomass in the biofilm. Three washings with sterile water were used to remove unbound colorant. Dissolution was performed by adding 200 µL of ethanol for 15 min, and, directly, the optical density (OD) was measured at 570 nm with BioTeK [54]. The anti-biofilm activity was calculated as follows:

Anti-biofilm activity (%) = (OD control − OD BS solution)/OD control × 100

### 4.7. DPPH Radical Scavenging Activity

The antioxidant activity of the biosurfactants was determined by colorimetric method based on their scavenging activities of 1,1-diphenyl-2-picrylhydrazyl (DPPH) free radicals [55]. For this, 1 mL of the BS dilution in methanol was added to 2 mL of 1,1-diphenyl-2-picrylhydrazyl methanolic solution (1:2). The mix reaction was shaken and incubated for 30 min in dark conditions. The color change indicated the presence of an antioxidant donator of hydrogen, reducing the DPPH radical to 2,2-diphenyl-1-picrylhydrazine (DPPH-H). The absorbance was measured at 517 nm. The scavenging activity was expressed as percentage of inhibition (I%) as follows:

I (%) = (Absorbance of Control − Absorbance of sample (BS)/Absorbance of control × 100

## 5. Conclusions

In summary, this work successfully identified new bacterial strain Zcb15, belonging to *Pantoea alhagi*. The strain Zcb15 produces glycolipids, identified by FTIR and GC/MS analyses, and showed high biosurfactant/bioemulsifier properties in the screening with multiple methods. The fatty acid composition of the cell membrane and BS were specifically related to this extremophile strain.

The BS described here possess antimicrobial and anti-biofilm behavior against human and plant pathogens and antioxidant effects. These findings promote their future use as a natural product for the pharmaceutical, food and agriculture fields.

## Figures and Tables

**Table 1:** Various vegetable oil effects on production of biosurfactants measured as UA/mL.

Vegetable Oil Types	Biosurfactant Activity (UA/mL) *
LB + oil corn	0.0152 ± 0.7
LB + sunflower oil	0.0202 ± 0.5
LB + Tween 80	0.0141 ± 0.48
LB olive oil	0.0151 ± 0.47

* Values are mean ± standard deviation of three triplicates.

**Table 2:** Fatty acid composition of the cell membrane of *Pantoea alhagi* and the secreted biosurfactants (BS).

Fatty Acid	Formula	BS	Cell Membrane
Methyl 2-hydroxydecanoate	2-OHC10:0	ND	0.46
Undecylic acid	C11	ND	0.02
Lauric acid	C12:0	ND	0.25
Methyl 2-hydroxydodecanoate	2-OHC12:0	ND	67.00
Tridecyl acid	C13:0	ND	0.09
Myristic acid	C14:0	0.21	0.00
Methyl 2-hydroxytetradecanoate	2-OHC14:0	0.44	2.63
Methyl 3-hydroxytetradecanoate	3-OHC14:0	ND	0.04
Methyl 13-methyltetradecanoate	i-C15:0	0.03	0.10
Methyl 12-methyltetradecanoate	a-C15:0	10.93	6.73
Palmitic acid	C16:0	0.26	0.06
Methyl 14-methylpentadecanoate	i C16:0	0.05	0.41
Methyl 2-hydroxyhexadecanoate	2-OHC16:0	0.05	0.07
Palmitoleic acid	C16:1n-9	0.03	0.02
Margaric acid	C17:0	ND	0.01
Methyl 14-methylhexadecanoate	i-C17:0	ND	12.05
Methyl cis-9,10-methylenehexadecanoate	C17:0d	ND	1.08
Stearic acid	C18:0	8.60	1.72
Oleic acid	C18:1n-9	76.26	3.06
Vaccenic acid	C18:1n-11	2.29	3.73
Nonadecylic acid	C19:0	0.05	0.12
Arachidic acid	C20:0	0.26	0.08

n-OH: hydroxyl group at C-n (n = 2, 3, 12…); i: branched-chain acid with branched methyl group at the iso position; a: branched-chain acid with branched methyl group at the anteiso position; C: carbon.

**Table 3:** Comparison of fatty acid ratios of different bacterial biosurfactants.

Strains [Ref]	Fatty Acid Composition (%)						
	C_13_	C_14_	C_15_	C_16_	C_18_	C_19_	C_20_
*Pantoea alhagi* [this work]	nd	(C14:0) 0.21	(a-C15:0) 10.93	(C16:0) 0.26	(C18:0) 8.6 (C18:1n-9) 76.26	(C19:9) 0.515	(C20:0) 0.26
*Bacillus subtilis* T89-15 [18,19]	3	2.4	67.7	22.7	3.4		
*Bacillus mojavensis* JF-2 [20]	1.3	49.6	11.3	24.4			
*Bacillus subtilis* sp. *spizizennii* T89-3 [18,19]	1.35	6.8	49.7	35.5	6.2		
*Buttiauxella* [21]		C14:0		C16:0	C18:0 C18:9		

**Table 4:** The antimicrobial activity of the biosurfactant pellet and the biosurfactants dissolved in ethyl acetate or methanol. Values expressed in mm present the diameter of the zone of inhibition as detected in the agar well diffusion test. Values are the average from triplicate experiments. Different subscript letters on the same line show significant difference at *p* < 0.05.

	Biosurfactants in Ethyl Acetate	Biosurfactants in Methanol	Biosurfactant Pellet
*Escherchia coli*	0 ± 0 ^b^	29.50 ± 1.00 ^a^	0 ± 0 ^b^
*Staphylococcus aureus*	23.75 ± 10 ^b^	36.00 ± 0.25 ^a^	0 ± 0 ^c^
*Micrococcus luteus*	16.00 ± 0.2 ^b^	15.75 ± 0.20 ^c^	21.75 ± 0.20 ^a^
*Fusarium* sp.	13.75 ± 0.57 ^c^	31.00 ± 0.50 ^a^	15.5 ± 0.5 ^b^

**Table 5:** The antioxidant activity of the biosurfactants secreted by *Panthoea alhagi* as compared to ascorbic acid (AA) expressed in percentage of inhibition of the free radical scavenging activity.

	DPPH (Inhibition %)
Biosurfactants in methanol (2 mg/mL)	78.07% ^a^
Ascorbic acid (0.5 mg/mL)	29.89 ^b^

^a,b^ Different subscript letters present significant difference at *p* < 0.05.

## Data Availability

Not applicable.

## Sample Availability

Samples of the compounds are available from the authors.
